# Dialectical Behavior Therapy (DBT) in an Assertive Community Treatment structure (ACT): treatment outcome of Integrated Care Borderline (ICB) in a two years follow-up

**DOI:** 10.1186/s40479-025-00321-3

**Published:** 2025-10-28

**Authors:** Hannah F. Warkentin, Julia Jegl, Katharina Krog, Buket Saricicek, Sarah V. Biedermann, Anne Karow, Jürgen Gallinat, Anja Zimmermann, Ingo Schäfer, Andreas Schindler

**Affiliations:** Department of Psychiatry and Psychotherapy, University Medical Center Hamburg-Eppendorf, Center for Psychosocial Medicine, Hamburg, Germany

**Keywords:** Integrated care borderline (ICB), Borderline personality disorder (BPD), Dialectical behavior therapy (DBT), Assertive community treatment (ACT)

## Abstract

**Background:**

We recently published treatment outcome data of patients with severe Borderline Personality Disorder (BPD) after one year of Dialectical Behavior Therapy (DBT) in Integrated Care Borderline (ICB). ICB provides DBT in the structures of an Assertive Community Treatment (ACT), working with a multi-professional outpatient team located in a psychiatric hospital. It integrates all elements of DBT with psychiatric and social support as well as crisis intervention if necessary. Previous data demonstrated significant improvements in BPD pathology and psychosocial functioning after one year. Since treatment typically took longer than one year, we now present data of the two years follow-up.

**Findings:**

In a sample of *N* = 31 patients with severe BPD outcome data after two years were compared to baseline data. Analyses show significant improvements in psychosocial functioning (GAF), BPD symptoms (BSL-23, SCID-II criteria), and other psychiatric symptoms (BSI, PHQ-9, PCL, suicidality), as well as a reduction of hospital days, and an increase in employment after two years of treatment. Effect sizes in most measures were medium to large, except psychiatric comorbidity (small effect) and anxiety (insignificant).

**Conclusion:**

Effect sizes after two years of ICB-treatment are larger than after one year, indicating an additional benefit of longer treatment duration for severely ill patients with BPD. Results further support the finding that DBT can be successful in outpatient settings and that ICB seems to have additional effects on employment and hospital days. The ICB approach appears to offer a viable framework for multi-professional outpatient DBT-teams.

**Trial registration:**

Registration number PV4920 - Integrated Care Borderline – Aerztekammer Hamburg, Germany.

## Introduction

ICB is a treatment model that is designed to treat persons with severe BPD in an outpatient setting. We recently published outcome data after one year of DBT treatment in ICB [1]. DBT has been shown to be effective in the treatment of BPD in inpatient as well as outpatient settings [2]. Due to insufficient treatment structures, these patients are often treated in inpatient settings. The ICB approach offers DBT within an ACT-framework. ACT models are usually based on multi-professional, community-based teams offering a variety of health care services for severely ill persons with psychosis and bipolar disorders. ICB adapts this approach for the treatment of patients with severe BPD. The core of ICB is a multi-professional outpatient team of psychotherapists, psychiatrists, and social workers. The team is located in a psychiatric hospital, using all of its facilities if needed. It provides all elements of DBT (individual therapy, skills training, telephone coaching, consultation team, supervision) and integrates psychiatric and social support as well as crisis interventions into a DBT-strategy [1]. After one-year of treatment, data showed significant improvements in clinical measures as well as a reduction of hospital days and an increase of employment. Effect sizes were small to medium [1]. Considering the severity of the disease, treatment often took longer than one year. To grasp the effects of these longer treatments we now present data of the two years follow-up. Additionally, this will make our data more comparable to other DBT trials, which typically report effects after two years [2].

## Method

### Study design

Data were collected in the course of the evaluation study of ICB [3] at the University Medical Center Hamburg-Eppendorf. This study ran parallel to the large RCT “RECOVER” [4], which was the background of the one-year analysis [1] and ended after one year. All participants gave their informed consent and were examined at the beginning of ICB treatment (Pre) and after two years of treatment (Post). The study was approved by the local ethics committee (Nr. PV4920).

### Participants

Patients with severe BPD were consecutively included in ICB treatment between 2015 and 2019. Inclusion criteria were a main diagnosis of BPD (ICD-10: F60.31) without excluding other comorbid psychiatric disorders except organic mental disorders (ICD-10: F0) and severe mental retardation (ICD-10: F72, F73). Participants had to be severely ill (Clinical Global Impression Scale (CGI) [6] >5; Global Assessment of Functioning (GAF) [5] <50.), they had to live within an 8 km range of the hospital and they needed sufficient language skills. Moreover, patients had to be insured with one of the participating health insurance companies. While the one-year analysis [1] was based on a sample of *N* = 50, the two-years-follow-up included all *N* = 31 patients, who were treated for two years. The decision to continue treatment for a second year was taken in a joined meeting of the consultation team with the patient at the end of the first year. Criteria were the progress of the patient in the first year and his or her need and commitment for further treatment. In *N* = 14 cases treatment was completed after one year, and *N* = 5 patients dropped out for different reasons (e.g. moving to another city, lack of motivation, not providing anymore research data, treatment of comorbid disorders in other facilities).

### Measures

Sociodemographic data as well as medical and treatment history were assessed at baseline only. All other data were assessed at baseline (Pre) and two years (Post) later. Psychosocial functioning was measured with the GAF [5]. The CGI [6] was used to map symptom severity. Borderline and other psychiatric symptoms were measured using the SCID-II-interview [7] and several self-report questionnaires: BPD symptoms using the Borderline Symptom List (BSL-23) [8], depressive symptoms using the Patient Health Questionnaire (PHQ-9) [9], anxiety symptoms with the Generalized Anxiety Disorder (GAD-7) [10], symptoms of posttraumatic stress disorder (PTSD) with the PTSD Checklist for DSM-5 (PCL-5) [11], and general symptoms with the Brief Symptom Inventory (BSI) [12]. Suicidality was assessed using a dichotomous item (suicidal/not suicidal in the last month), according to the SCID-II-criterion covering suicidal ideation as well as suicidal behavior. Hospital days were operationalized as days spent in psychiatric day care or inpatient treatment in the last twelve months prior to Pre and in between Pre and Post assessments. Employment status was assessed by the Location & Vocation Index (VILI) [13] and dichotomized into “employed” (working, in education) and “not employed” (sick, unemployed, retired).

### Statistical analysis

Differences in the variables over two years were analyzed using *t*-tests for paired samples and Wilcoxon signed-rank tests depending on the distribution of the data. Accordingly, Cohen’s *d* and *r* are reported to display effect sizes. Changes in the proportion of suicidal and (un-)employed individuals between Pre and Post were analyzed using McNemar’s test. As an intent-to-treat sample, missing values in baseline and follow-up data were imputed according to the expectation maximization algorithm and used in the statistical analysis. The two-sided type I error was set to α = 5%. All analyses were performed in an exploratory manner without adjustment for multiplicity. Analyses were performed using IBM SPSS Statistics 25.

## Results

### Sample

Baseline sociodemographic and clinical characteristics can be found in Table 1. Most of the sample was female, single, and unemployed. The baseline mean GAF score was below 40, which is lower than the cut-off of severe mental illness. Most participants had harmed themselves, a majority had tried to commit suicide in the past, and on average, they had spent more than three of the last 12 months in psychiatric hospitals. Compared to the sample at the one-year follow-up [1], this sample shows a slightly lower GAF score, as well as slightly higher rates of suicide attempts, self-harm, and hospital days, suggesting that the more severely ill individuals were treated for two years.

**Table 1:** Baseline sociodemographic and clinical characteristics of the sample

Variables	ICB treatment group (*n* = 31)
Age (years, mean (SD))	26.2 (6.8)
Sex (female, n (%))	26 (83.9)
Marital status (single, n (%))	20 (64.5)
School graduation (n (%))	29 (93.5)
Vocational status (unemployed, n (%))	20 (64.5)
Comorbid disorders	
Comorbid mental disorder (yes, n (%))	31 (100)
Comorbid somatic disorder (yes, n (%))	22 (71.0)
Treatment at baseline (n (%))	
Inpatient (hospital)	8 (25.8)
Day clinic (hospital)	2 (6.5)
Outpatient	13 (41.9)
No treatment	7 (22.6)
GAF score (mean (SD))	39.8 (5.5)
CGI score (mean (SD))	5.7 (0.7)
Hospital days in the last 12 months (mean (SD))	89.8 (75.2)
Suicidal attempt in the past (yes, n (%))	18 (58.1)
Self-harm in the past (yes, n (%))	28 (90.3)
Age of disorder onset (mean (SD))	13.4 (5.6)
Age of initial psychiatric contact (mean (SD))	14.7 (9.9)

### Outcome

Psychosocial functioning (GAF) significantly improved over the two-year treatment period (*p* <.001), with a large effect size (*r* =.80). Self-reported BPD symptoms (BSL-23) decreased significantly *t*(30) = 2.82, *p* =.008, 95%-*CI* [0.18, 1.09], with a medium effect size of *d* = 0.51. Similarly, BPD criteria assessed via external rating (SCID-II) significantly declined between Pre and Post (*p* <.001), with a large effect size (*r* =.72). Depressive symptoms (PHQ-9) improved significantly over the treatment period *t*(30) = 2.94, *p* =.006, 95%-*CI* [1.12, 6.19], with a medium effect size (*d* = 0.53). Anxiety symptoms (GAD-7) did not show significant reductions across time *t*(30) = 1.75, *p* =.090, 95%-*CI* [−0.37, 4.91]. PTSD symptoms (PCL-5) significantly decreased after two years of treatment *t*(30) = 4.63, *p* <.001, 95%-*CI* [9.01, 23.39], with a large effect size (*d* = 0.83). General psychiatric symptoms (BSI) showed significant reductions across the measurement time points *t*(30) = 2.05, *p* =.049, 95%-*CI* [0.001, 0.68], with a small effect size of *d* = 0.37. Hospital days decreased from 90 days in the year before ICB to 5 days in the two years ICB-treatment, resulting in a large effect (*r* =.78; *p* >.001). The proportion of patients who were currently suicidal decreased significantly (*p* =.002). The proportion of patients who were employed increased significantly (*p* =.039) (Table 2).

**Table 2:** Differences after two years of ICB treatment

Differences after 24 months of treatment					
Variable	Baseline (Pre; M (SD))	Two years (Post; M (SD))	Observed difference (M (SD))	*p*	Effect size d/*r*
Psychosocial functioning (GAF)	39.77 (5.51)	49.01 (8.36)	9.24 (8.52)	**< 0.001**	*r* =.80
Borderline symptoms (BSL-23)	2.22 (0.84)	1.59 (1.04)	−0.63 (1.25)	**0.008**	d = 0.51
Borderline symptoms (SCID-II)	7.65 (1.48)	5.19 (2.02)	−2.45 (2.57)	**< 0.001**	*r* =.72
Depressive symptoms (PHQ-9)	16.70 (5.39)	13.05 (6.58)	−3.65 (6.91)	**0.006**	d = 0.53
Anxiety symptoms (GAD-7)	12.75 (4.59)	10.48 (5.46)	−2.27 (7.20)	0.090	d = 0.32
PTSD symptoms (PCL)	41.92 (16.14)	25.69 (16.39)	−16.23 (19.51)	**< 0.001**	d = 0.83
Overall psychiatric symptoms (BSI)	1.77 (0.74)	1.43 (0.79)	−0.34 (0.92)	**0.049**	d = 0.37
Hospital days	89.84 (75.17)	5.13 (11.79)	−84.71 (73.41)	**< 0.001**	*r* =.78

Variable	Baseline (Pre; %)	Two years (Post;%)	Observed difference (%)	*p*	
Current suicidality (yes)	29 (93.5)	19 (61.3)	10 (33.3)	**0.002**	
Employment status (ES; employed)	11 (35.5)	18 (58.1)	7 (22.6)	**0.039**	

*p*-values in bold face indicate significance

## Discussion

This study aimed at evaluating a model of Integrated Care for patients with severe BPD over a treatment period of two years. The sample presented here is severely ill with impairments in psychiatric, somatic, and social dimensions. It is slightly more severely impaired part of the sample the sample described at one year follow-up [1].

After two years of treatment in ICB significant improvements in almost all symptoms examined can be shown: while GAF scores increased, BPD, depressive, PTSD, psychiatric symptoms in general and suicidality decreased. Compared to the one-year analysis [1] data show further improvements and larger effect sizes in several areas, especially in BPD-pathology (BSL-23; *r* =.51 vs. *r* =.31) and psychosocial functioning (GAF; *r* =.80 vs. *r* =.64). The only symptom area without significant reductions over time was anxiety. Since DBT addresses several symptoms that are related to anxiety, this finding will need further attention in future analyses. However, our data confirm two important results of the one-year analysis. Hospital days showed a sharp decrease from 90 days in the year before ICB to five days per year during ICB treatment. Additionally data confirm the significant increase in employment reported in the one-year analysis.

## Conclusions

Our data support the finding that it is possible to treat patients with severe BPD successfully with DBT in an outpatient ACT-structure. Two years of ICB-treatment show similar medium to large effects as comparable studies [2, 14, 15] and larger effects compared to one year of treatment. Though in the absence of a control group we cannot draw any causal conclusions, the advantage of the ICB treatment structure over other settings might be the additional effect on hospital days and employment.

### Limitations

While the one-year analysis was conducted as a part of a large RCT (RECOVER) [1, 4], the two-year analysis was not controlled. Future studies will have to cover longer periods and will have to work with RCT-designs, to be able to draw more reliable conclusions on the effects of ICB in the long run. Sample size was small, relying only on patients who were treated for two years, resulting in low statistical power. We did not run any corrections for multiple testing, possibly leading to an overestimation of effects. Suicidality was only assessed as a one-item dichotomous variable according to SCID-criteria. The reduction of hospital days and the increase of employment indicate reductions in treatment costs and social costs. Unfortunately, costs were not assessed.

## Data Availability

The datasets used and/or analyzed during the current study are available from the corresponding author on reasonable request.

## Abbreviations

ACT: Assertive Community Treatment
BPD: Borderline Personality Disorder
BSI: Brief Symptom Inventory
BSL-23: BSL-23 Borderline Symptom List-23
CGI: Clinical Global Impression Scale
DBT: Dialectical Behavior Therapy
GAD-7: Generalized Anxiety Disorder 7
GAF: General Assessment of Functioning
ICB: Integrated Care Borderline
PHQ-9: Patient Health Questionnaire 9
RCT: Randomized Controlled Trial
SCID: Structured Clinical Interview for DSM Disorders
SMI: Severe Mental Illness
